# Sleep for Young People

**Published:** 1892-01

**Authors:** 


### SLEEP FOR YOUNG PEOPLE.

A German specialist, Dr. Cold, has recently pleaded for giving young people more sleep. A healthy infant sleeps most of the time during the first weeks ; and in the early years people are disposed to let children sleep as much as they will. But from six or seven, when school begins, there is a complete change. At the age of ten or eleven the child sleeps only eight or nine hours, when he needs at least ten or eleven ; and, as he grows older, the time of rest is shortened. Dr. Cold believes that up to twenty a youth needs nine hours' sleep, and an adult should have eight or nine. With insufficient sleep, the nervous system, and brain especially, not resting enough, and ceasing to work normally, we find exhaustion, excitability, and intellectual disorders gradually taking the place of love of work, general well being, and the spirit of initiative.

